# Changes of neuregulin-1(NRG-1) expression in a rat model of overactive bladder induced by partial urethral obstruction: is NRG-1 a new biomarker of overactive bladder?

**DOI:** 10.1186/1471-2490-13-54

**Published:** 2013-10-23

**Authors:** Hoon Jang, Dong Seok Han, Seung Mo Yuk

**Affiliations:** 1 The Department of Urology, The Catholic University of Korea, DaeJeon St. Mary’s Hospital, Daeheung-dong, jug-gu, Daejeon, South Korea

**Keywords:** Neuregulin-1, Overactive bladder, Biomarker

## Abstract

**Background:**

To determine whether neuregulin-1(NRG-1) is a potential new biomarker of overactive bladder (OAB) induced by partial urethral obstruction in a rat model of OAB and to evaluate the urothelium as a therapeutic target of OAB.

**Methods:**

Female Sprague–Dawley rats were separated into three 20-animal groups: normal, OAB, and 5-hydroxymethyl tolterodine (5-HMT)-treated OAB. In the OAB and OAB + 5-HMT groups, the urethra of each animal was partially obstructed; the OAB + 5-HMT group received intravenous 5-HMT for 3 weeks. At the conclusion of the 5-HMT dosing, the rats in each group underwent cystometrography, and the bladders were histologically evaluated. The expression of brain derived-neurotrophic factor (BDNF) and NRG-1 were evaluated in the urothelium.

**Results:**

Compared with the control group, the OAB group showed a markedly increased bladder weight and a significant decrease in the micturition interval and volume; rats in the OAB + 5-HMT group showed decreased bladder weights and an improved micturition interval and volume. BDNF and NRG-1 were expressed at significantly higher levels in the OAB group, and were significantly reduced in the OAB + 5-HMT group compared with the control group.

**Conclusions:**

The study suggests that NRG-1 is a potential new biomarker of OAB; the urothelium might be a therapeutic target for OAB treatment.

## Background

Overactive bladder (OAB) is characterized by the presence of urinary urgency, with or without urge incontinence, usually accompanied by daytime frequency and nocturia, in the absence of proven infection or other obvious pathology [1]. Urinary urgency, defined as a sudden compelling desire to void that is difficult to defer, is the unique symptom that must be present in order to establish the diagnosis of OAB [2]. Multiple questionnaries (e.g., USS and OABq) are widely used for quantifying and grading urgency severity [3,4] because the diagnosis of OAB is currently based on the presence of subjective symptoms. However, objective tests to diagnose OAB and assess therapeutic outcomes are urgently needed.

Until recently, the best way to objectively diagnose OAB was to measure detrusor overactivity (DO) because DO is the urodynamic hall mark of OAB. However, urodynamic test are invasive and can only identify OAB in half of the patients, as normal individuals often have asymptomatic, involuntary detrusor contractions [5]. These facts decrease the usefulness of this test as a tool for diagnosing OAB. To improve the diagnosis of OAB, recent studies have focused on the detection and clinical application of OAB biomarkers. Some studies have indicated that nerve growth factor (NGF), brain-derived neurotrophic factor (BDNF), prostaglandins, cytokines, and C-reactive protein may be suitable biomarkers of OAB [6].

NRG-1 is one of 4 neuregulin proteins that act on the epidermal growth factor receptor (EGFR) family of receptors [7] and is produced in numerous isoforms by alternative splicing, allowing it to perform various functions [8,9]. Acetylcholine receptor-induced activity (ARIA), an alternative name for type 1 NRG-1, plays a role in synapse development, influencing the upregulation of acetylcholine receptor genes beneath the endplate after mammalian motor neurons have made synaptic contact with muscle fibers. In this study, we focused on the changes in NRG-1 in a rat model of OAB and evaluated its potential as an OAB biomarker.

## Methods

### Animal groups and treatment protocol

Sixty, 12-week-old female, Sprague–Dawley rats (250–300 g, Daehan Biolink, Daejeon, Korea) were used. The experimental protocol was approved by the Animal Ethics Committee of the University of Chungnam, South Korea (CNU-00289) and conducted according to the National Institutes of Health (USA) guidelines. Rats were separated into three, 20-animal groups: control, OAB, and OAB + 5-hydroxymethyl tolterodine (5-HMT). All rats in the OAB and OAB + 5-HMT groups underwent partial urethral obstruction (PUO) and those in the control group underwent sham operations. Rats in the OAB + 5-HMT group also received 0.1 mg/kg of 5-HMT (Pfizer, Sandwich, UK) via the tail vein (2 times/week) over 3 weeks. These doses corresponded to the doses clinically used in humans [10]. Cystometrography (CMG) was performed on all rats 4 weeks after PUO.

### Partial urethral obstruction (PUO)

PUOs were created as described by Melman *et al.*[11]. Briefly, the bladder of each anesthetized rat was approached, and the proximal urethra exposed, through a lower midline incision. A 3–0 polypropylene suture was used to tie the urethra with a 24-G angioneedle sheath. After suturing, the angioneedle sheath was removed, leaving the urethra partially obstructed. In our experiment, two rats in the OAB group died during the surgeries and one rat in the OAB + 5-HMT group also died after operation.

### Cystometrography (CMG)

The catheter implantation procedures needed for CMG were completed 4 days before the evaluation. A cuffed polyethylene tube (PE20, A-M Systems, Carlsberg, WA, USA), was inserted through an abdominal incision into the dome of the bladder and held in place with a purse-string suture. During CMG, the end of tube was connected to a pressure transducer (Powerlab, ADInstrument, Sydney, Australia) and an infusion pump (Promed-Tech., Bellingham, MA, USA) via a 3-way stopcock, to record intravesical pressure (IVP) and to infuse saline into the bladder. Micturition volumes (MVs) were recorded using a fluid collector connected to a force displacement transducer (Grass Instruments, Quincy, MA, USA). Room-temperature saline was continuously infused into the bladder at a rate of 0.5 mL/min. After the voiding pattern stabilized, the micturition cycles were recorded. IVP and MV were recorded synchronously and continuously using a Chart v 5.5.6 for Windows data acquisition system (AD Instrument) at a sampling rate of 2000 Hz.

### Tissue preparation for analysis

After CMG, all rats were euthanized, and each animal’s bladder was excised at the level of the proximal urethra and weighed. For routine histological analyses, the bladders of some animals in each of group were fixed overnight with 4% paraformaldehye at 4°C. The fixed tissue was sectioned and stained with hematoxylin and eosin before being evaluated by a pathologist. The bladders from most animals were dissected free of the urothelium using microscissor and microforceps. Each sample was stored at −70°C until needed. The urothelium was used for reverse transcriptase-polymerase chain reaction (RT-PCR) analyses.

### Reverse transcriptase-polymerase chain reaction

Total RNA was extracted from a frozen urothelium sample by adding 1 mL of TRIzol (Invitrogen, Carlsbad, CA, USA) to each sample and homogenizing the tissue in a 5-mL glass tube. The homogenate was transferred to a 1.0-mL tube and mixed with 0.2 mL of 99% chloroform (Sigma-Aldrich, St. Louis, MO, USA). After incubating for 5 min at room temperature, the homogenate was centrifuged for 10 min (13,200 × g) at room temperature. The supernatant was transferred to a clean tube and 1 mL of absolute isopropyl alcohol (Sigma-Aldrich) was added, followed by centrifugation. The supernatant was discarded, and the cell pellet was mixed with 0.5 mL of diethylpyrocarnobate (DEPC, Sigma - Aldrich), and centrifuged for 10 min (13,200 × g) at room temperature. After the supernatant was discarded, the pellet was dried at room temperature, dissolved with DEPC-treated water, and stored at −75°C. Agarose gel electrophoresis and ethidium bromide staining, followed by visual examination under ultraviolet light, was used to assess the quality and integrity of the recovered RNA samples. cDNA was prepared using a first-standard cDNA synthesis kit (Enzynomics, Yuseo, Korea), according to the manufacturer’s protocol. The cDNA was incubated at 50°C for 50 min, 70°C for 10 min, and finally stored at −20°C.; the glyceraldehydes-3-phosphate dehydrogenase (GAPDH) gene was used as reference standard. After an initial denaturation step at 95°C for 5 min, 35 annealing cycles (58°C for 30 s), and an extension procedure (72°C for 30 s and 72°C for 5 min) were performed. PCR products were separated on 1.2% agarose gels containing ethidium bromide, visualized, photographed on an ultraviolet transilluminator, quantified by densitometry, and qualified by Bio-ID (Vilber Lourmat, Torcy, France).

### Data analysis

Data were statistically compared between the 3 groups using one-way analysis of variances (ANOVA), followed by Scheffe’s multiple comparison post-hoc test. Comparisons between 2 groups were performed using Student’s *t*-test. Differences were considered statistically significant when *P* < 0.05, with results expressed as the mean (SD).

## Results

### Bladder weight and histological examination

Table 1 shows the body and bladder weights for the 3 groups; the mean body weights for the 3 groups were not significantly different (*P* = 0.691). However, the mean weights of the bladders in the OAB group were significantly higher than in the control group (*P < 0.001*); there were also significant weight differences between the OAB and OAB + 5-HMT bladders (*P < 0.001*).

**Table 1:** Changes in body and bladder weights in the experimental groups

	**Control**	**OAB**	**OAB + 5-HMT**
**Body Weight (g)**	228.13 ± 13.601	229.92 ± 10.727	231.65 ± 13.258
**Bladder Weight (mg)**	116.53 ± 8.262	212.08 ± 13.883^※^	197.43 ± 13.737^※※^

*OAB* overactive bladder group; *OAB + 5-HMT* overactive bladder treated with 5-hydroxymethyl tolterodine group.

^※^ Significant difference (P < 0.05) compared with the control group.

^※※^ Significant difference (P < 0.05) compared with OAB group.

**Figure 1:**
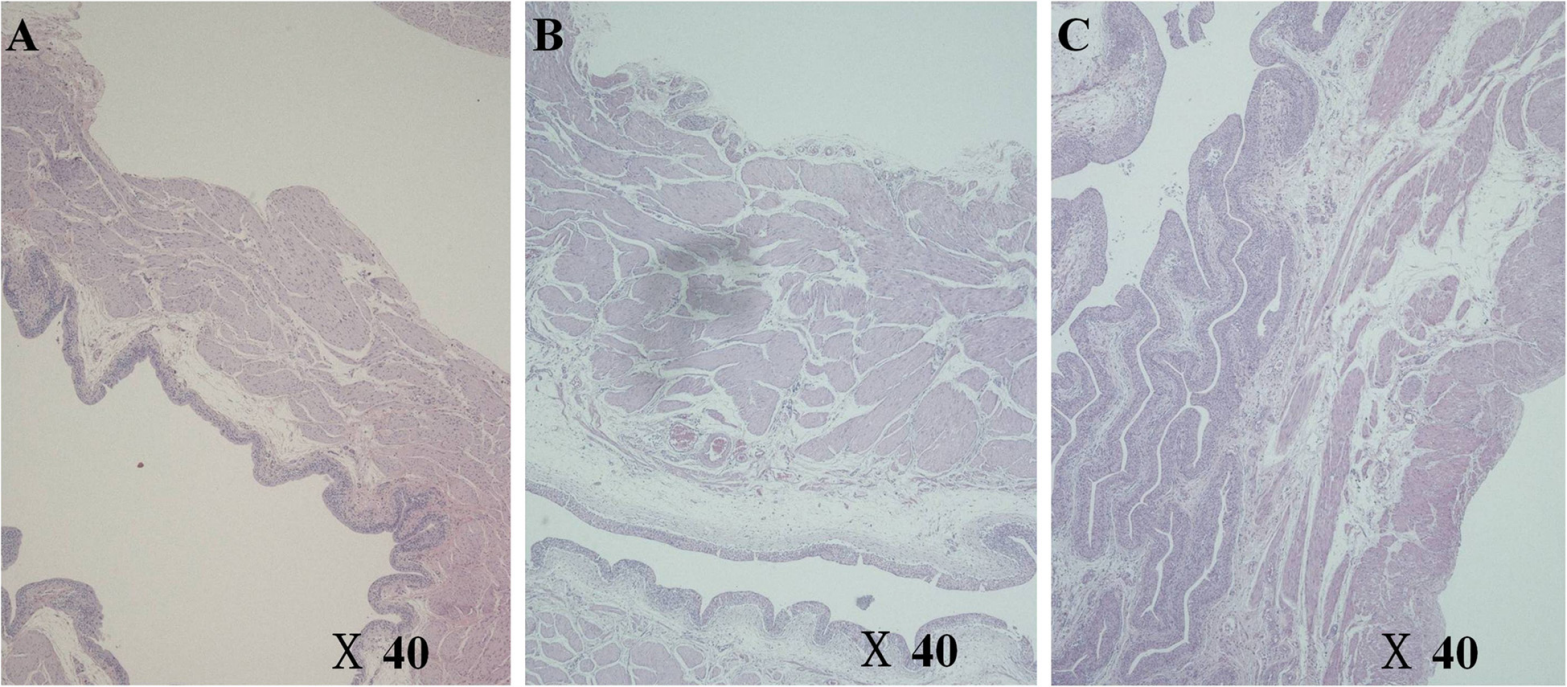
**Hematoxylin and eosin staining of bladder tissue.** The muscular layer appears more hypertrophic in image **B** and **C** than in image **A**. There was no typical difference in the urothelium between the groups. **A**, Control group; **B**, overactive bladder group; **C**, overactive bladder treated with 5-hydroxymethyl tolterodine group.

In histological examinations, detrusor muscle hypertrophy seemed more extensive in the OAB and OAB + 5-HMT groups than in the control group. However, there was no typical urothelium difference between groups (Figure 1).

### *In vivo* assessment of bladder function

**Figure 2:**
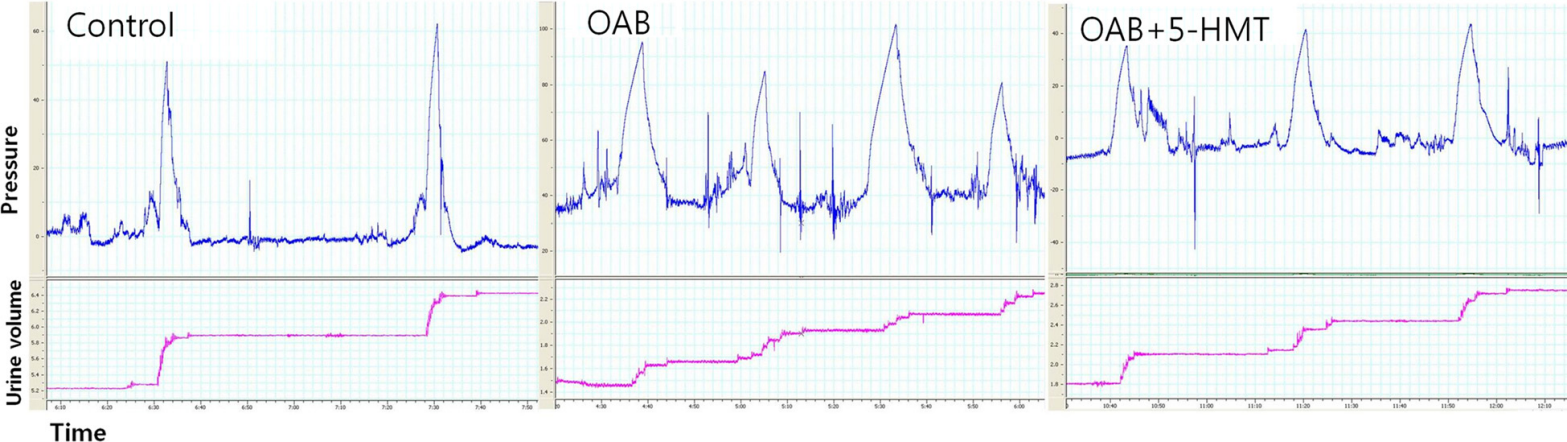
**Typical cystometrograms of the experimental groups.** Micturition interval and micturition volume were decreased in the OAB group compared with the control group. The OAB + 5-HMT group demonstrated improvements compared with the OAB group. Involuntary contractions, not observed in the control group, were observed during the micturition interval in OAB group. OAB, overactive bladder group; OAB + 5-HMT, overactive bladder treated with 5-hydroxymethyl tolterodine group.

Table 2 describes the micturition intervals (MIs), pressure differences between the maximal micturition pressure and the basal pressure (MP-BP), micturition volumes (MVs), and the micturition times (MTs) during CMG. Figure 2 shows a representative CMG tracing. CMG revealed significant decreases in the MIs (*P* < 0.001) and MVs (*P* < 0.001) in the OAB animals compared with the controls. Furthermore, 5-HMT administration had a significant effect on MIs (*P* < 0.001) and MVs (*P* < 0.001), compared with the OAB group. The mean MT was significantly higher in the OAB group (*P* = 0.010) than in the control group. However there was no statistical difference between the OAB and OAB + 5-HMT groups (*P* = 0.923); significant differences in MP-BP were not observed among the 3 groups (*P* = 0.547).

**Table 2:** Results of cystometric analysis of the experimental animals

	**Control**	**OAB**	**OAB + 5-**
			**HMT**
**Micturition Interval**	64.73 ± 4.68	27.97 ± 4.19^※^	39.25 ± 6.02^※※^
**(Sec) (Mean ± SD)**			
**Pressure Difference between Maximal Micturition Pressure and Basal Pressure (cmH2O) (Mean ± SD)**	55.35 ± 4.55	50.79 ± 8.40	48.17 ± 4.99
**Micturition Volume**	0.612 ± 0.610	0.186 ± 0.049^※^	0.270 ± 0.655^※※^
**(ml) (Mean ± SD)**			
**Micturition Time**	10.63 ± 0.83	11.73 ± 1.20^※^	10.78 ± 1.49
**(Sec) (Mean ± SD)**			
**Pressure Difference between Threshold Pressure and Basal Pressure**	13.93 ± 1.05	9.47 ± 6.97	4.74 ± 3.74
**(cmH2O) (Mean ± SD)**			

*OAB* overactive bladder group; *OAB + 5-HMT* overactive bladder treated with 5-hydroxymethyl tolterodine group.

^※^ Significant difference (P < 0.05) compared with the control group.

^※※^ Significant difference (P < 0.05) compared with OAB group.

### Comparison of growth factors in the urothelium

**Figure 3:**
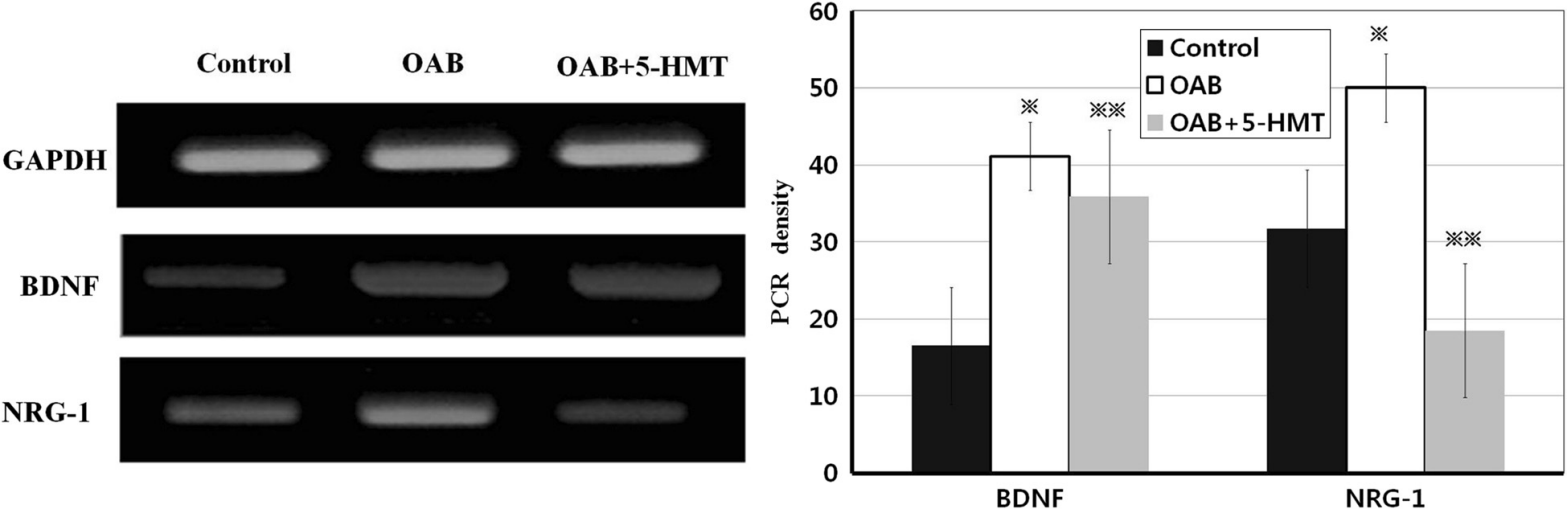
**The reverse transcription-polymerase chain reaction for the presence of urothelial growth factors.** OAB, overactive bladder group; OAB + 5-HMT, overactive bladder treated with 5-hydroxymethyl tolterodine group. ※ Significant difference (P < 0.05) compared with the control group. ※※ Significant difference (P < 0.05) compared with OAB group.

RT-PCR was used to assess the expression of BDNF and NRG-1 in urothelium of animals from each group (Table 3). Figure 3 shows the representative intensity differences for growth factors in the experimental groups. Expression of BDNF and NRG-1 was significantly higher in the OAB group than in the control group (*P < 0.001*). However, the expression of BDNF and NRG-1 was significantly less in the OAB + 5-HMT group than in the OAB group (*P < 0.001*).

**Table 3:** Growth factors density comparisons among the experimental groups

**Value**	**Control**	**OAB**	**OAB + 5-HMT**
**GAPDH**	124.94 ± 0.13	125.14 ± 0.49	124.87 ± 0.79
**NRG 1**	31.73 ± 0.28	50.02 ± 0.67^※^	18.52 ± 0.02^※※^
**BDNF**	16.53 ± 0.13	41.14 ± 0.21^※^	35.91 ± 0.56^※※^

*OAB* overactive bladder group; *OAB + 5-HMT* overactive bladder treated with 5-hydroxymethyl tolterodine group.

^※^ Significant difference (P < 0.05) compared with the control group.

^※※^ Significant difference (P < 0.05) compared with OAB group.

## Discussion

The diagnosis of OAB is symptom-based and involves assessing the conscious perception of urinary urgency. Knowing whether an animal is experiencing urgency, even if pseudoaffective changes in behavior may suggest it, is impossible. Animals cannot relate their symptoms to investigators, and consequently, the creation of an animal model of OAB has not been technically possible. Nevertheless several animal model of OAB, involving partial bladder outlet obstruction, spontaneous hypertension, hyperlipidemia, various neurological insults, and some gene knock-outs, have been suggested and used to study the mechanisms and treatment of OAB [12].

In this study, we used a partial bladder outlet obstruction animal model. This model has advantages in the replication of many of the structural and physiological bladder changes seen in human bladder outlet obstruction and is known to be a reliable model with good etiological validity [12]. The main findings of the present study were: (1) bladder weights increased after PUO, but there was a no apparent histological change in urothelial layer; (2) DO, induced by PUO, was identified by CMG; (3) BDNF and NRG-1 expression levels were significantly higher in the OAB group than in the control group and significantly lower in the OAB + 5-HMT group than in the OAB group.

In this work, detrusor muscle hypertrophy was histologically observed in the OAB and OAB + 5-HMT groups. Although some reports have suggested that detrusor wall thickeness is a potential marker of OAB [13], other studies have suggested that detrusor wall thickness is not a diagnostic tool for OAB. Nevertheless, detrusor hypertrophy is associated with detrusor overactivity and it is important hallmark of reproducing OAB in animal model. Instead of measuring detrusor wall thickness, we evaluated bladder weight because the histology of the bladder tissue was inconsistent among the groups. Based on these results, we suggest that the increased bladder weight represents detrusor muscle hypertrophy because the detrusor muscle composes most of the bladder wall and 5-HMT is effective in decreasing detrusor hypertrophy.

DO is a urodynamic observation characterized by involuntary contractions during filling phase of cystometry [1]. However, DO is not an essential characteristic of OAB, as indicated by a 2006 study that found only 64% of diagnosed OAB patients, according to the new International Continence Society (ICS) definition, had urodynamically demonstrable DO [5]. The study also showed that more than 30% of DO patients did not have OAB. Nonetheless, DO has been used as the core surrogate for urgency in basic OAB research as urinary urgency cannot be ascertained in animal models of OAB [12]. During CMG in the present study, MIs and MVs were significantly decreased and involuntary contractions were present during the MIs in the OAB group. The decreased MIs and MVs were thought to correspond to the clinical the frequent and involuntary contractions during MI.

Although the exact mechanism of OAB is not completely understood, OAB may be related to changes in or dysfunction of the muscarinic receptors of the detrusor muscle; other mechanisms may also be involved that include other receptor systems [14,15]. These changes result in a predisposition towards unstable bladder contractions or overactivity of the detrusor muscle. Thus, the major mechanism of anticholinergic drugs, widely used in treatment of OAB, is their antagonism of the effect of acetylcholine on muscarinic receptors in the cholinergically innervated bladder detrusor muscle. The result is a decreased number of detrusor smooth muscle contractions in the bladder and a reduced intensity of urgency symptoms [16,17]. In this study, we used 5-HMT, the active metabolite of fesoterodine, for treating experimental OAB; fesoterodine is a widely used muscarinic receptor antagonist for treating OAB, in humans [18]. In our study, the bladder weights, MIs and MVs were improved in the OAB + 5-HMT group, compared with the OAB group, suggesting that the changes may be due to the anti-cholinergic effect of 5-HMT.

In this study, we also isolated the urothelium from the bladder and estimated BDNF expression as a control marker of NRG-1 for evaluating NRG-1’s potential as an OAB biomarker. OAB has been suggested to be related to neurotransmitters and other receptor systems in the urothelium. Furthermore, these sensor molecules and malfunctioning of the urothelium may be linked to the development of OAB [19-22]. The urothelium is increasingly recognized as a responsive structure capable of detecting physiological and chemical stimuli, and of releasing several signaling molecules and various trophic factors after physical or chemical stimulation. Thus, the functioning of the urothelium is closely related to the functioning of the nervous system, and control the urothelium signaling pathway may be a new therapeutic target for treatment of OAB.

As described in the Introduction, new, noninvasive tests to diagnose OAB and assess therapeutic outcomes are urgently needed and some studies have focused on the detection and clinical application of OAB biomarkers [23]. In this study, we presumed that elevated bladder pressure, caused by PUO, stimulated the urothelium, and increased the release of neuronal molecules, such as BDNF and NRG-1. These neuronal molecules were also presumed to activate a signaling pathway for inducing detrusor muscle hypertrophy and overactivity. The expected result was an increase in the number of detrusor smooth muscle contractions and an increased intensity of urgency symptoms. To confirm this hyposthesis, we measured the expression of BDNF and NRG-1 in isolated urothelium tissues.

In humans, the BDNF protein is encoded by the *BDNF* gene [24]. BDNF is a member of the neutrophin family of growth factors, which are related to nerve growth factor. BDNF is the most abundant neurotrophin in the human body and contributes to the differentiation, survival and normal function of sensory neurons [25-28]. Many studies have suggested that BDNF is a biomarker of OAB because urinary bladder synthesis of BDNF is strongly increased after chronic bladder inflammation or spinal cord injury [29-31]. BDNF sequestration improves bladder function in rats with chronic cystitis [32], and the BDNF/creatinine ratio is significantly higher in OAB patients compared to controls [33]. Furthermore, urinary concentrations of BDNF are higher at baseline than after administration of botulinum toxin A to the bladder trigone in patients with bladder pain syndrome/interstitial cystitis [34]. These positive correlations suggest the potential utility of BDNF as a biomarker of OAB.

In humans, NRG-1 protein is encoded by the *NRG-1* gene [7,35] and is a member of the epidermal growth factor (EGF) family known to activate proliferation, differentiation, and survival of many tissue types [7,36-38]. Although the function and mechanism of NRG-1 has not yet been clearly established, at least 6 major types (having different N termini) of NRG-1 are known [39]. Type I NRG-1 plays a particular role in synapse development, influencing the upregulation of acetylcholine receptor genes beneath the endplate after mammalian motor neurons have made synaptic contact with muscle fibers. ErbB4 is highly expressed during re-epithelialization of urothelium [40], and antiadrenergic NRG-1/ErbB signaling disappears when the muscarinic cholinergic receptor is blocked [41]. In addition, antiadrenergic muscarinic cholinergic signaling is diminished in the absence of NRG-1 [42]. Although the molecular mechanism underlying the cooperation between the NRG-1/ErbB system and the cholinergic system are still under investigation, NRG-1 may be related to the recycling and maintenance of urothelium and may act in a manner similar to a cholinergic receptor in the urothelium.

In this study, both BDNF and NRG-1 were expressed at significantly higher levels in the OAB group, compared with the control group, and were significantly reduced in the OAB + 5-HMT group compared to the OAB group. As a result, we suggest that the increased expression of NRG-1 induced upregulation of the acetylcholine receptors, including detrusor muscle hypertrophy and increasing the involuntary contraction of the detrusor smooth muscle. Furthermore, NRG-1 expression may be altered by the anti-cholinergic drug, 5-HMT, which affected not only the muscarinic receptor in the detruosor muscle but also the muscarinic receptor in the urothelium. Therefore, the urothelium may be a therapeutic target for treatment of OAB.

We note that our study has some limitations. In this study, we compared NRG-1 expression in control, OAB, and OAB + 5-HMT groups without investigating the function and detailed mechanism of NRG-1 action in the urothelium. Had we assessed the signaling NRG-1 pathway in the bladder urothelium, better information about the function and mechanism of NRG-1 could have been provided, along with additional evidence for its consideration as an OAB biomarker. However, NRG-1 has not been studied in urological organ or disease models and our study was designed to investigate the possibility NRG-1’s role as a potential OAB biomarker. To clarify NRG-1’s role as a potential OAB biomarker, further investigations regarding the relationship between NRG-1 and the bladder urothelium signaling pathways are needed.

## Conclusions

These results suggest that NRG-1 is a potential biomarker of OAB in rat OAB models. The results also suggest that the urothelium is a coordinator, rather than a simple receptor that detecting physical or chemical stimuli, and may release several signaling molecules, making the urothelium signaling pathway a potential therapeutic target for OAB treatment.

## Abbreviations

NRG-1: Neuregulin-1; OAB: Overactive bladder; 5-HMT: 5-hydroxymethyl tolterodine; BDNF: Brain derived-neurotrophic factor; DO: Detrusor overactivity; PUO: Partial urethral obstruction; CMG: Cystometrography.

## Competing interests

The authors declare that they have no competing interests.

## Authors’ contributions

Dr. HJ conducted the animal study and drafted a paper. Dr. DSH participated in the animal study and in the drafting of the manuscript. Dr. SMY designed the study and supervised the writing of the manuscript. All authors read and approved the final manuscript.

## Pre-publication history

The pre-publication history for this paper can be accessed here:

http://www.biomedcentral.com/1471-2490/13/54/prepub
